# Genome-Wide Identification of Rare and Common Variants Driving Triglyceride Levels in a Nevada Population

**DOI:** 10.3389/fgene.2021.639418

**Published:** 2021-03-02

**Authors:** Robert W. Read, Karen A. Schlauch, Vincent C. Lombardi, Elizabeth T. Cirulli, Nicole L. Washington, James T. Lu, Joseph J. Grzymski

**Affiliations:** ^1^Center for Genomic Medicine, Desert Research Institute, Reno, NV, United States; ^2^Department of Microbiology and Immunology, School of Medicine, University of Nevada, Reno, Reno, NV, United States; ^3^Helix Opco, LLC., San Mateo, CA, United States; ^4^Renown Health, Reno, NV, United States

**Keywords:** GWAS, PheWAS, triglycerides, whole exome sequencing, rare variant analysis

## Abstract

Clinical conditions correlated with elevated triglyceride levels are well-known: coronary heart disease, hypertension, and diabetes. Underlying genetic and phenotypic mechanisms are not fully understood, partially due to lack of coordinated genotypic-phenotypic data. Here we use a subset of the Healthy Nevada Project, a population of 9,183 sequenced participants with longitudinal electronic health records to examine consequences of altered triglyceride levels. Specifically, Healthy Nevada Project participants sequenced by the Helix Exome+ platform were cross-referenced to their electronic medical records to identify: (1) rare and common single-variant genome-wide associations; (2) gene-based associations using a Sequence Kernel Association Test; (3) phenome-wide associations with triglyceride levels; and (4) pleiotropic variants linked to triglyceride levels. The study identified 549 significant single-variant associations (*p* < 8.75 × 10^–9^), many in chromosome 11’s triglyceride hotspot: *ZPR1*, *BUD13*, *APOC3*, *APOA5*. A well-known protective loss-of-function variant in *APOC3* (R19X) was associated with a 51% decrease in triglyceride levels in the cohort. Sixteen gene-based triglyceride associations were identified; six of these genes surprisingly did not include a single variant with significant associations. Results at the variant and gene level were validated with the UK Biobank. The combination of a single-variant genome-wide association, a gene-based association method, and phenome wide-association studies identified rare and common variants, genes, and phenotypes associated with elevated triglyceride levels, some of which may have been overlooked with standard approaches.

## Introduction

Hypertriglyceridemia is prevalent in the US adult population: 31% have borderline high triglyceride measurements (≥150 mg/dL) and 16% have high triglyceride levels (≥200 mg/dL) (Miller et al., 2011). Severe hypertriglyceridemia (≥500 mg/dL) was shown to be associated with 33–38% greater medical costs in 2008, after adjustment of clinical conditions such as cardiovascular disease, heart failure, hypertension, and diabetes (Nichols et al., 2011). Elevated triglycerides are associated with diabetes (Subramanian and Chait, 2012) and a reported risk factor for coronary heart disease (CHD), yielding an ongoing national health concern (Kathiresan et al., 2008; Ewald and Kloer, 2012; TG and HDL Working Group of the Exome Sequencing Project et al., 2014; Han et al., 2016; Siewert and Voight, 2018).

Hypertriglyceridemia is a notable health burden in Nevada, where many residents live in rural, typically underserved communities, including more than thirty unique Native American tribal reservations. Contribution to this health concern is the high percentage of adult obese and overweight Nevadans (27 and 66%, respectively), as it is well known that body mass index (BMI) is linked with blood triglyceride levels (Kim et al., 2012; Van Hemelrijck et al., 2018). The Healthy Nevada Project (HNP), a population health study developed in collaboration between Renown Health and the Desert Research Institute in Reno, Nevada, was established in 2016 to examine the effects genetics may have on Nevadan health outcomes. Whole-exome sequencing data paired with cross-referenced Electronic Health Records (EHR) are now available for more than 30,000 participants in Northern Nevada. Although many studies have examined the effects between single nucleotide variants and triglyceride levels (Kathiresan et al., 2008; Lippi et al., 2008; Coram et al., 2013; Weissglas-Volkov et al., 2013; TG and HDL Working Group of the Exome Sequencing Project et al., 2014; Dron and Hegele, 2017; Yamada et al., 2018), none, to the best of our knowledge, have performed a three-pronged approach: single-variant genome-wide association studies (GWAS) of both rare and common variants; gene-based association analysis of both common and rare variants (Ionita-Laza et al., 2013); and comprehensive phenome-wide analyses (PheWAS) (Carroll et al., 2014). Via this approach, our study replicated a number of well-known triglyceride-linked variants and identified several variants with no known associations to triglycerides. Both the single-variant GWAS and the gene-based association results were validated in a cohort (*N* = 35,321) of UK Biobank participants with exome-sequencing (Van Hout et al., 2019). Lastly, comprehensive EHR-based phenome-wide analyses uncovered clinical conditions associated with changes in triglyceride levels and examined pleiotropy in triglyceride-linked variants.

This triglyceride-focused study examines common, rare, and very rare variants on a genome-wide and phenome-wide scale.

## Materials and Methods

### Data Disclosure Statement

In order to minimize unintentional sharing of information that can be used to re-identify private information, a subset of the phenotype data generated for this study is available at https://www.dri.edu/renown-ihi/healthynvprojectgenetics/. Additionally, genotype data that support the findings of this study will be made available upon reasonable request. Please see Data Availability Statement.

### The Renown EHR Database

The Renown Health EHR system was instantiated in 2007 on the EPIC system (EPIC System Corporation, Verona, WI, United States), and contains lab results, diagnosis codes (ICD9/ICD10), and sociodemographic information of approximately 1.6 million hospital patient visits from 2005 to the present date.

### Genotype Sample Collection

The HNP is a population health study of Nevadans, with specific targeted recruitment in rural and socioeconomically depressed Northern Nevada areas. The project consists of two phases: Phase I began in 2016, in which genotyping was conducted on 10,000 adult volunteer participants as described in Read et al. (2019); Schlauch et al. (2020). Phase II was initiated in 2018, using the Helix Exome+ platform (Helix, San Diego, CA, United States). As of December 2020, approximately 30,000 sequenced Phase II participants in the HNP have cross-referenced electronic medical records. The study presented here examines a subset of 9,183 European HNP participants with at least two recorded triglyceride and BMI measurements. We refer to this as the HNPT_EU cohort.

### IRB and Informed Consent

This study was conducted under a human subject protocol approved by the University of Nevada Institutional Review Board under project #1106618-15. Participants in the Healthy Nevada Project undergo written and informed consent to having genetic information associated with electronic health information (EHR) in a de-identified manner. Inclusion criteria are individuals older than 18 years who can appear in person at an HNP study location to participate in the education and consent process. A copy of the consent can be found at https://healthynv.org/about/consent/. Patient identifiers are not incorporated into the research EHR: the EHR and genetic data are linked in a separate environment via a unique identifier as approved by the IRB.

### Processing of Clinical Data

Most HNPT_EU cohort participants had multiple BMI recordings across the 14 years of EHR; the mean number of BMI records across the individuals was 16.2 records; the maximum number of BMI measures per individual was 652. For HNPT_EU individuals with more than one recorded BMI measure, a more complex quality control step was first performed before computing the average BMI value to remove likely erroneous values. Specifically, if participant *i* had multiple BMI records, the standard deviation σ_*iBMI*_ of those records was computed. If any of participants’ BMI measures were less than the threshold σ*_*T*1_* or greater than the threshold σ*_*T*2_* (explained below), they were excluded before computing the average of the remaining BMI measures. The threshold σ*_*T*1_* is the lower 2.5th percentile of the approximately normal distribution of; similarly, σ*_*T*2_* is its upper 2.5th percentile. This additional quality control step excluded BMI values such as “3986.19” and “3.” A total of 4.7% of outlying BMI values for those individuals with multiple records were excluded.

The majority of HNPT_EU participants also have multiple triglyceride lab results recorded across the 14 years; these results span nine different lab tests each with independent reference ranges. We standardized these reference ranges using methods from Read et al. (2019). This approach applies a simple linear transform to convert each test’s reference range into the most commonly recorded test. For individuals with multiple triglyceride records, outliers were also excluded following the same process as with multiple BMI measures described above. Mean quality-controlled BMI and triglyceride values for the HNPT_EU cohort, Type II diabetes ICD 9/ICD 10 codes and antihyperlipidemic medications are available at the link: https://www.dri.edu/renown-ihi/healthynvprojectgenetics/.

### Sequencing

Sequencing was performed in the Helix Laboratory (CLIA #05D2117342, CAP# 9382893) using the Helix Exome+, a proprietary medical-grade exome that includes additional non-coding targets resembling a microarray backbone within one sequencing assay (Helix., 2019; Cirulli et al., 2020). Coverage for this platform is based on 4,000 Exome+ results (2,000 male and 2,000 female) and includes full base-pair level histograms (Helix., 2019). Results demonstrate that more than 90% of the bases have greater than or equal to 20× coverage for popular reference panels including ACMG-59 and the Ashkenazi Jewish carrier screen. Moreover, this assay has been validated using high confidence calls from public reference materials such as the Platinum genomes (Eberle et al., 2017) and the National Institute of Standards and Technology (NIST) Genome in a Bottle (GIAB) (Zook et al., 2014) with sensitivity, precision, repeatability, and reproducibility all greater than 99.9%. Sequencing data were aligned to GRCh38 with variant calling implemented by Helix. (2019) and Kendig et al. (2019) following established sequencing-specific quality control metrics and GATK best practices (Helix., 2019; Cirulli et al., 2020).

### Statistical Analysis of Sequencing Data

Raw genotype data of rare and common variants were processed through quality control pipelines for a single-variant GWAS modified to include rare variants (Anderson et al., 2010; Panoutsopoulou and Walter, 2018; Cirulli et al., 2020), using GRCh38. Relationship inference was performed with KING, which identified 4,019 pairs of first-degree relatives (Manichaikul et al., 2010). For all related participants, only the participant with the highest genotyping rate was retained (Anderson et al., 2010). Variants out of Hardy–Weinberg equilibrium (*p* < 1 × 10^–6^) were excluded. Genotype call rates were similar to those in other studies (Reed et al., 2015; Panoutsopoulou and Walter, 2018). Quality control thresholds were as follows: variant call rates greater than 95% and individual call rates greater than 70% were deemed high quality. To ensure statistically powerful rare-variant associations, any variant with less than ten copies of the minor allele across the HNPT_EU cohort was removed. This resulted in 5,712,318 non-pruned, high-quality variants in the single-variant GWAS. Variants are generally classified as rare if their minor allele frequency (MAF) < 0.01; low-frequency when 0.01 ≤ MAF < 0.05; and common if MAF ≥ 0.05. The filtered high-quality variants contained 35.84% rare, 20.26% low-frequency, and 43.89% common variants. To distinguish the many rare variants in our platform with MAF as low as 0.0002, we use the term “very rare” variants with 0.0002 < MAF < 0.001. Sequencing ontologies were noted for all variants (i.e., missense, nonsense, synonymous, indels, frameshifts, etc.), and no variants were excluded based on ontology type.

Variants underwent the same quality control in the SKAT gene collapse as for the single-variant analysis above, and those in linkage disequilibrium (LD) with surrounding variants were pruned. *PLINK* v1.9 (Purcell et al., 2007) was implemented for pruning, using standard parameters (50 variants per sliding window; window size of five variants; *r*^2^ = 0.5) (Anderson et al., 2010). As an additional quality control step for the gene-based analysis, variants with less than three copies of the minor allele were excluded. Variants carried by one or two people may, in some cases, indicate a sequencing error and may decrease the strength and specificity of the analysis. Note that gene-based methods are better-powered, so the previous threshold of a minimum of five carriers in the single-variant GWAS was relaxed. This removed 5,459,154 variants from the analysis. A total of 2,697,018 variants was used in the discovery gene collapse analysis: 69.96% were rare, 11.68% were low-frequency, and 18.36% were common. These variants were grouped into 25,283 gene sets.

A standard principal component analysis (PCA) applied to pruned variants corrected for population substructure in the single-variant, gene-based, and phenotype-wide association analyses. Statistical models were adjusted by the first four genotypic principal components (P1, P2, P3, and P4), which yielded a genomic inflation factor of λ ≤ 1.003.

### Effect Sizes

Effect sizes are measured here as raw (non-standardized) beta-coefficients of the genotype covariate in the linear regression model for each variant using *PLINK*. Sizes are reported in terms of triglyceride measures in mg/dL units, as in Willer and Mohlke (2012). Note that power studies performed on standardized effect sizes of the linear regression are identical to those performed on raw values. By standardizing the response variable (e.g., triglyceride measures) into its *z*-score and then performing linear regression, the transformed genotype effect size is in terms of standard deviation units of the original response variable (Supplementary Text).

### Genotype Annotation

Variants were annotated generally using dbSNP build 153^1^ and PhenoScanner V2 (Staley et al., 2016; Kamat et al., 2019). Functional characterization of variants was performed by Ensembl Variant Effect Predictor v.101 (VEP) (Supplementary Table 1). Subsequently, single-variant and gene-based associations were mapped using PhenoScanner V2, ClinVar (January 31, 2021^2^), and the NHGRI-EBI Catalog of human genome-wide association studies^3^. If associations or their proxies were not found in these databases, they are denoted in the manuscript as “not published to the best of our knowledge.”

### Power of Single-Variant GWAS

QUANTO (Gauderman, 2002) was applied to approximate necessary sample sizes to detect a range of effect sizes with several MAFs and at least 80% statistical power under the additive model at a two-sided Type I error level of 5%. The mean and standard deviation triglyceride levels of the HNPT_EU cohort were used (119.29 and 67.64 mg/dL, respectively). To detect standardized effect sizes greater than one standard deviation unit with adequate power and reasonable sample sizes, the MAF of a single variant was restricted to at least 5 × 10^–4^; tests of variants with this MAF were sufficiently powered to detect standardized effect sizes of one standard deviation (67.64 mg/dL) with a sample size of *N* = 9,183. Note that this MAF corresponds to approximately ten carriers in a sample of size 9,183. Similarly, tests of variants with MAF of 0.001 are well-powered to detect effect sizes of 42.5 mg/dL; tests of variants with MAF of 0.005 are adequately powered to detect effect sizes of 20 mg/dL, both with *N* = 9,183. Variants with MAF between 0.01 and 0.05 generate well-powered hypothesis tests to detect effect sizes between 14 and 6.5 mg/dL, respectively, with *N* = 9,183. Tests of common variants proved powerful enough to test much smaller effect sizes: any test with common variants is well-powered to detect effect sizes less than 6.5 mg/dL with 9,183 participants. These observations show that the HNPT_EU of 9,183 participants allows for adequately powered tests of both rare and common variants in the single-variant GWAS, with larger and smaller effect sizes, respectively.

### Single-Variant Genome-Wide Association Study (GWAS)

Using 9,183 participants with high-quality call rates, a linear regression on triglyceride levels vs. genotype was performed with *PLINK* v1.9, under the additive genetic model with covariates age, sex, BMI, DM2 diagnosis, whether the participant was on an antihyperlipidemic drug at any time, and the first four genotypic principal components (PC1–PC4). This model represents a combination of statistical models used in previous association studies targeting triglyceride measures (Teslovich et al., 2010; Willer et al., 2013; Gao et al., 2014; Davis et al., 2017). Age was defined as the participant age in 2020. Residuals were normally distributed under this model (Hoffmann et al., 2018b). Association tests with *p*-values less than 8.75 × 10^–9^ were considered statistically significant: a threshold based on a Bonferroni correction with the total number of high-quality variants used in the GWAS.

### Gene-Based Collapse Analysis

SKAT is a standard method to examine gene effects by combining common and rare variants in a gene by up-weighting rare variants and down-weighting common variants to balance effect sizes (Lee et al., 2012; Ionita-Laza et al., 2013). The SKAT analysis was performed using the R package SKAT^4^, and the weighting scheme outlined by Ionita-Laza et al. (2013): rare variants are weighted as *Beta*(MAF; 1,25); common variants are weighted as *Beta*(MAF; 0.5,0.5). Covariates included in the model were the same as those used in single-variant GWAS: age, sex, BMI, DM2 diagnosis, whether the participant was on an antihyperlipidemic drug at any time, and PC1–PC4. Gene-based association tests with *p*-values less than 2.0 × 10^–6^ were considered statistically significant; this threshold was based on a Bonferroni correction with *n* = 25,283 (Auer and Lettre, 2015).

### Phenome-Wide Association Study (PheWAS)

**FIGURE 1:**
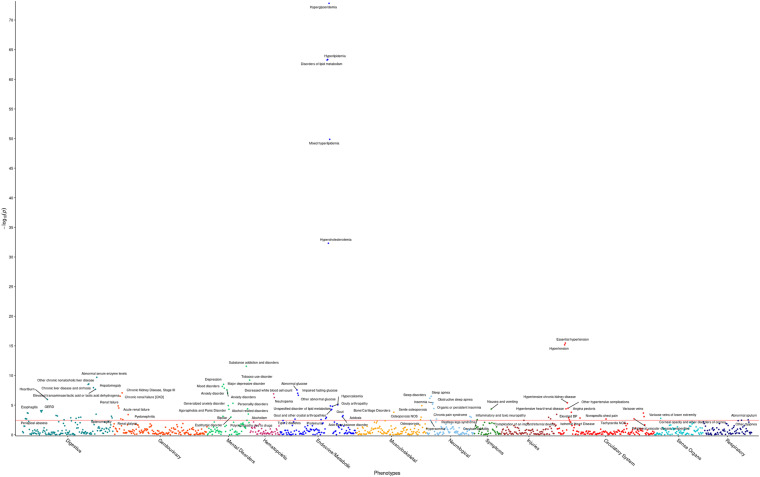
Phenome-wide analysis in the HNPT_EU between triglyceride levels and EHR diagnoses. Each point represents the *p*-value of an individual association between triglyceride levels and incidence of one of 1,372 phenotype groups, with covariates age, sex, DM2, and PC1–PC4. The *x*-axis shows different phenotypic conditions, grouped into 11 groups. The *y*-axis presents the -log_10_ transform of the *p*-value of each association. The significance level α = 3.8 × 10^– 3^ is shown by the horizontal red line. Comprehensive results can be found in Supplementary Table 2.

The R package PheWAS v. 0.99.5.4 (Carroll et al., 2014) was used as a basis to perform phenome-wide association analyses (PheWAS). Specifically, the PheWAS investigated whether triglyceride levels are a predictor of incidence of specific phenotype groups in the HNPT_EU. Each phenotypic group was investigated via a simple logistic regression using the covariates age, sex, BMI, DM2 diagnosis, and the first four genotypic principal components to adjust for ethnicity. Participant ICD codes recorded in the EHR were converted into 1,857 phenotype groups (“phecodes”) using the PheWAS package as described in Denny et al. (2013) and Carroll et al. (2014); 1,386 of these phenotype groups contained more than 20 cases and were examined for association with triglyceride levels. Any model in which the maximum likelihood estimate could not be calculated due to quasi-complete or complete separation by the predictor variable (Venables and Ripley, 1999) was excluded at this point from further investigation. The significance level was computed by first calculating the adjusted *p*-values for the multiple hypothesis tests performed using the Benjamini–Hochberg false discovery rate (FDR) (Benjamini and Hochberg, 1995) and selecting the raw *p*-value corresponding to the FDR = 0.05 significance level, following a modification of Denny’s protocol (Denny et al., 2013). This level (α = 3.8 × 10^–3^) is represented by the red line in Figure 1. Statistically significant results are shown in Supplementary Table 2.

A series of PheWAS were performed to investigate pleiotropy of the 549 variants found to be statistically significant in the single-variant GWAS. Specifically, each analysis examined 760,914 possible phenotype–genotype associations of 549 variants and 1,386 phenotype groups with at least 20 cases in the HNPT_EU. In order to identify associations across diverse conditions and diseases, three statistical models were used: the identical model as in the GWAS; a second model broadened to include age, sex, BMI, and PC1–PC4; and a third model including only age, sex, and PC1–PC4. These two latter models directly follow published studies that identify pleiotropy in triglyceride-associated variants (Ridker et al., 2008; Dehghan et al., 2011; Kraja et al., 2011; Middelberg et al., 2011; Schunkert et al., 2011; Nelson et al., 2017; Hoffmann et al., 2018b; Ligthart et al., 2018). The additive genotype model was used as the predictor for the phenotype. To account for spurious results due to either the small counts of rare variants in case/control cohorts, or possible diagnosis or data-entry errors in the EHR, a conservative Bonferroni correction was performed to adjust for the false discovery rate.

Any model in which the maximum likelihood estimate could not be calculated due to quasi-complete or complete separation of the phenotype incidence by minor allele incidence (Venables and Ripley, 1999) was excluded from further investigation. The significance level of each PheWAS was then computed via a Bonferroni correction as α = 0.05/*N*, with *N* the number of feasible models tested. Models with the predictor (genotype) coefficient deemed statistically different to zero via a *t*-test at a *p*-value less than α were retained. A standard Fisher exact test (one degree of freedom) was performed on the allelic distribution between cases and controls as in Schlauch et al. (2016, 2019). The raw *p*-value and power of the Fisher test are included in Supplementary Table 3. The table also contains the allelic odds ratio that describes the association between the specific phenotype and minor allele of each variant, irrespective of covariates. The final column (Supplementary Table 3) indicates which covariates were included in the statistical model.

### Biobank Validation

The UK Biobank Resource (UKB^5^) provided a validation cohort for HNPT_EU single-variant and gene-based association discoveries. The validation cohort includes 35,321 European UKB participants with BMI measures [Field ID 21001], triglycerides [Field ID 30870], incidence of DM2 [Field ID 130708], and antihyperlipidemic medications [BNF code in the GP prescription records, Field ID 42039]. UKB participants had one or two records of the continuous variables; thus means were computed without any further quality control. We used the FE version 43 of the UKB *PLINK*-formatted exome files [Field ID 23160] (Regier et al., 2018) for the gene-based analysis. Imputed UKB genotypes [Field ID 22801–22823] were used to replicate HNPT_EU associations. Demographics of the UKB cohort are presented in Supplementary Table 4.

Quality control steps for UKB exome data closely followed that of the HNPT_EU Exome+ data platform. Related individuals were removed using the genetic kinship matrix provided by the UKB [Field ID 22021]. Variants out of Hardy–Weinberg equilibrium (*p* < 1 × 10^–6^) were excluded. Empirical calculations of the UKB differed due to the difference in sequencing platforms; call rate distributions were used to set thresholds: call rates less than 99% and individual call rates less than 98% were removed. *PLINK* v1.9 (Purcell et al., 2007) removed variants in LD, based on the same parameters used in the discovery cohort.

Single-variant validation included the identical linear model as in the discovery cohort with covariates age, sex, BMI, DM2 diagnosis, antihyperlipidemic medication status, and the first four principal components. Principal components were generated identically to those in the HNP cohort. The same significance level was used (*p*-value < 8.75 × 10^–9^). Gene-based validation in the UKB was based on 1,935,811 single variants and its significance level was identical to the discovery analysis (*p* < 2.0 × 10^–6^).

## Results

### Demographics

The mean level of standardized and quality-controlled triglyceride levels of the HNPT_EU was 119.29 mg/dL with standard deviation of 67.64 mg/dL (Table 1). As the effect size is directly related to the expected value (mean) and variation of the response variable, we expect raw effect sizes to be proportionally large values.

**TABLE 1:** Demographic information for HNPT_EU.

Cohort size	9,183
Age (years)	58.68 ± 15.62
Male (%)	3,081 (33.55)
DM2	1,489 (16.21)
Prescribed antihyperlipidemics	3,544 (38.59)
Quality-controlled BMI	29.13 ± 6.43
Quality-controlled triglycerides	119.29 ± 67.64

**Table of cohort characteristics. Continuous variables are presented as mean ± SD; categorical variables are presented as counts and percentages. All values were standardized with a custom algorithm to remove outliers (see section Materials and Methods). BMI is in kg/m^2^; triglyceride units are mg/dL*.*

### Single-Variant GWAS

The single-variant GWAS identified 549 statistically significant non-pruned variants (*p* < 8.75 × 10^–9^) associated with triglyceride levels in the HNPT_EU (Supplementary Figure 1 and Supplementary Table 5). As Supplementary Figure 1 shows, 144 variants in the well-known triglyceride hotspot 11q23.3 were shown to be associated with strong significance to triglyceride levels in the HNPT_EU. There are published associations within this multi-gene region (*BUD13*, *ZPR1*, *APOC3*, *SIK3*, and *APOA5*), as well as *MLXIPL, LPL*, and *GCKR* to triglyceride levels: we list only a few here (Kathiresan et al., 2008; Willer et al., 2008; Hegele et al., 2009; Hindorff et al., 2009; Murray et al., 2009; Ariza et al., 2010; Johansen et al., 2010; Ken-Dror et al., 2010; Teslovich et al., 2010; Waterworth et al., 2010; Kraja et al., 2011; Comuzzie et al., 2012; Kettunen et al., 2012; Coram et al., 2013; Keller et al., 2013; Lutz et al., 2015; Yamasaki et al., 2015; Hoffmann et al., 2018b; van der Harst and Verweij, 2018; Wojcik et al., 2019).

### Gene-Based Association Analysis

**FIGURE 2:**
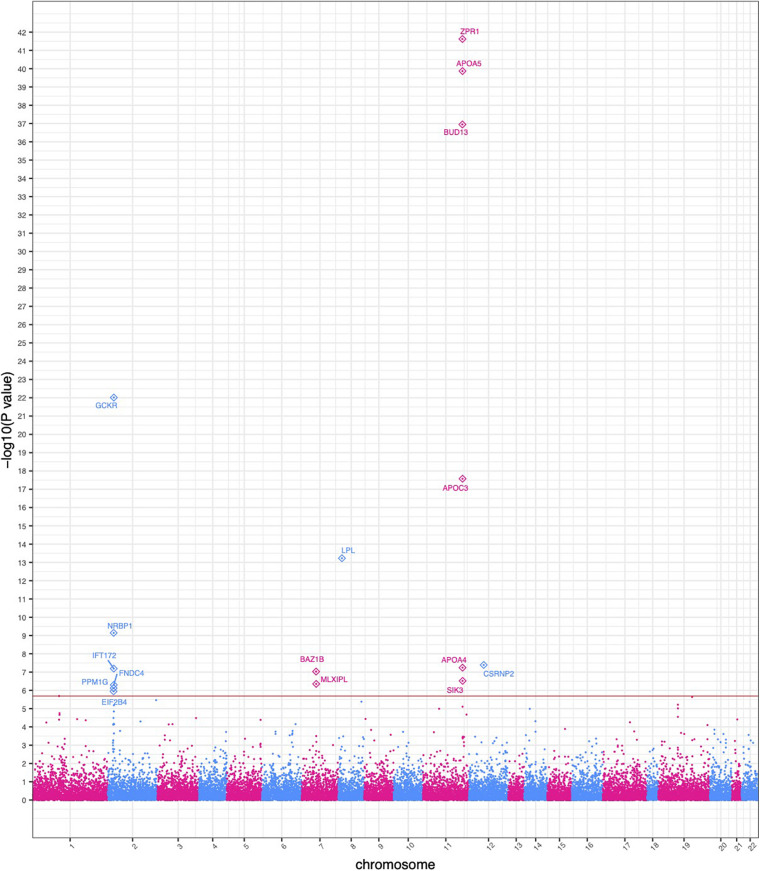
Manhattan plot of significant SKAT-based gene collapse results. The *x*-axis represents the genomic start position of 25,283 genes. The *y*-axis represents -log_10_-transformed raw *p*-values of each genotypic association. For ease of viewing, only genes above the horizontal line, which indicates the significance level α = 2.0 × 10^– 6^, are annotated.

SKAT identified 16 gene-based associations at a significance level of α = 2.0 × 10^–6^ (Figure 2 and Table 2) in the HNPT_EU. Fifteen of these were previously published as being related to triglyceride, lipid and/or BMI measurements including *APOA5*, *ZPR1, BUD13*, *APOC3, GCKR*, *LPL, SIK3, BAZ1B*, and *APOA4* (Merkel et al., 2002; Tsutsumi, 2003; Mar et al., 2004; Florvall et al., 2006; Corella et al., 2007; Willer et al., 2008, 2009, 2013; Hegele et al., 2009; Hindorff et al., 2009; Murray et al., 2009; Qi et al., 2009; Ariza et al., 2010; Delgado-Lista et al., 2010; Johansen et al., 2010; Keebler et al., 2010; Ken-Dror et al., 2010; Waterworth et al., 2010; Chambers et al., 2011; Kraja et al., 2011; Major et al., 2011; Ota et al., 2011; Carvalho-Wells et al., 2012; Coram et al., 2013; Weissglas-Volkov et al., 2013; Hassan et al., 2014; Yamasaki et al., 2015; Lu et al., 2016; Obata et al., 2016; Sakamoto et al., 2018) (Supplementary Table 6). Several of these genes reside in the chromosome 2 and 11 triglyceride hotspots. The two most significant gene-based associations in the HNPT_EU were the zinc finger protein encoded by *ZPR1* and Apolipoprotein A-V encoded by *APOA5* (*p* = 2.39 × 10^–42^ and *p* = 1.36 × 10^–40^, respectively) (Table 2).

**TABLE 2:** Significant gene-based associations.

Gene Id	Chrom	Gene contains at least one significant variant from the single-variant GWAS	The gene collapse association *p*-value is greater than the single-variant association *p*-value for all variants in the gene	HNPT_EU gene-collapse *p*-value	UKB gene-collapse *p*-value	HNPT_EU test statistic	HNPT_EU number of tested variants	HNPT_EU number of rare variants tested	HNPT_EU number of common variants tested	Previous association with triglycerides
ZPR1	11	Y	N	2.39 × 10^–42^	1.17 × 10^–75^	66.88	34	26	8	*
APOA5	11	Y	Y	1.36 × 10^–40^	1.16 × 10^–124^	52.05	25	19	6	*
BUD13	11	Y	N	1.13 × 10^–37^	2.82 × 10^–53^	48.04	65	34	31	*
GCKR	2	Y	N	9.75 × 10^–23^	1.26 × 10^–38^	35.39	73	57	16	*
APOC3	11	Y	Y	2.65 × 10^–18^	2.69 × 10^–85^	28.54	18	13	5	*
LPL	8	Y	Y	5.93 × 10^–14^	1.51 × 10^–69^	22.09	106	72	34	*
NRBP1	2	Y	N	7.15 × 10^–10^	2.33 × 10^–15^	19.50	31	29	2	*
CSRNP2	12	Y	N	4.09 × 10^–08^	9.35 × 10^–01^	14.46	32	25	7	
APOA4	11	N	*NA*	5.73 × 10^–08^	2.01 × 10^–35^	14.14	30	20	10	*
IFT172	2	N	*NA*	6.46 × 10^–08^	7.48 × 10^–14^	17.96	118	92	26	*
BAZ1B	7	N	*NA*	9.38 × 10^–08^	8.45 × 10^–15^	15.34	97	67	30	*
SIK3	11	Y	N	3.03 × 10^–07^	1.92 × 10^–12^	13.61	321	175	146	*
MLXIPL	7	Y	N	4.45 × 10^–07^	1.97 × 10^–37^	13.44	81	56	25	*
FNDC4	2	N	*NA*	5.03 × 10^–07^	6.35 × 10^–12^	14.72	10	9	1	*
PPM1G	2	N	*NA*	7.58 × 10^–07^	9.36 × 10^–01^	11.99	37	29	8	*
EIF2B4	2	N	*NA*	1.14 × 10^–06^	1.05 × 10^–17^	12.06	36	26	10	*

**This table shows the statistically significantly associated genes with triglyceride levels in the HNPT_EU and UKB as identified by the SKAT gene-based association analysis. The second column indicates whether the gene contains a variant identified in the single-variant GWAS: this demonstrates whether a gene association would have been undetected based on the single-variant analysis alone. The third column indicates whether the gene-based association is stronger than any single variant association in the gene: “N” indicates that one single-variant association may be driving the gene-based association; “Y” indicates that a combination of variants is driving the association. Genes marked with an “^∗^” in the last column have a published association with triglycerides. References for those associations can be found in Supplementary Table 6*.*

Ten of the significant gene-based associations contained at least one statistically significant variant identified by the single-variant GWAS. Three of these genes had a stronger agglomerative gene-based effect than their respective single-variant associations; thus, their associations were not driven by a single statistically significant variant (Table 2). Conversely, six gene-based associations had no overlap in the single-variant GWAS. These associations would be undetected in a study based solely on a single-variant GWAS.

### Significant Phenotypic Associations With Triglyceride Levels

The first PheWAS identified expected statistically significant phenotypic associations of triglyceride levels. Triglyceride levels were strong predictors of incidence of hyperglyceridemia, hyperlipidemia, disorders of lipid metabolism, hypertension, and hypercholesterolemia, in tandem with the covariates. Additionally, triglyceride levels demonstrated a significant positive effect in ischemic heart disease; hypertensive heart, renal, and kidney disease; chronic liver disease; a number of mental disorders, as well as sleep disorders. Higher triglyceride levels showed a protective effect on osteoporosis. Comprehensive results are shown in Figure 1 and Supplementary Table 2.

### Pleiotropy of Rare and Common Variants

The second series of PheWAS identified variants with a significant association to one or more phenotype groups. Many of the variants were associated with hyperglyceridemia, supporting both EHR diagnoses and the GWAS results above. One example of recurring pleiotropy is the rare missense variant rs137891079, which is a significant predictor of higher triglyceride levels in the HNPT_EU, and is also shown to be a predictor of cerebral atherosclerosis. Additionally, this variant was found to be involved in osteomyelitis, and other infections within and outside of the bone. The variant is not published in current releases of PhenoScanner, NHGRI-EBI GWAS Catalog or ClinVar. We note that the variant is extremely rare, with only 10 carriers in the HNPT_EU, of which two are in the cerebral atherosclerosis case group. The variant rs947056517 is shown to be associated with increased incidence of HNPT_EU pancreatic disease; it is also extremely rare and is not currently published with any association to the best of our knowledge. Results from each model are presented in Supplementary Table 3.

### UK Biobank Validation

Significant single-variant associations identified in the HNPT_EU were examined for validation in the UKB: results are presented in Supplementary Table 7. Approximately 50% of the significant HNPT_EU variants lie on chromosomes 8 and 11 and were used for validation. Of 264 significant variants, 253 (95.8%) are imputed genotypes in the UKB resource. Of these, 209 (82.6%) were validated at *p* < 8.75 × 10^–9^. The effect direction of all variants was equivalent between the two cohorts, and most had similar magnitudes. Table 2 outlines the validation of gene-based association results between the two cohorts. Of 16 genes, 14 (87.5%) were validated in the UKB with increased significance. The two genes that did not demonstrate significant association to triglycerides in the UKB cohort were *PPM1G* and *CSRNP2*.

## Discussion

### Single-Variant GWAS

Variants in well-known triglyceride-related genes such as *BUD13* and *SIK3* are known to be associated with triglyceride levels, lipid traits, overall lipid homeostasis, or metabolic syndrome (Kathiresan et al., 2008; Kraja et al., 2011; Keller et al., 2013; Sakamoto et al., 2018). Other expected single-variant associations include those in *ZPR1*, which codes for a regulatory protein known to bind several transcription factors that may influence obesity (Ueyama et al., 2015); variants within *ZPR1* are known to affect triglyceride levels, as well as modulate HDL and total cholesterol levels (Comuzzie et al., 2012; Hoffmann et al., 2018b; Wojcik et al., 2019). The gene *ZPR1* is known to interact with the triglyceride-associated gene *APOA5* (Ueyama et al., 2015), which plays an important role in regulating plasma triglyceride levels, a major risk factor for coronary artery disease (CAD) (van der Harst and Verweij, 2018). Variants in *APOA5* are associated with triglyceride levels, diseases involving lipid traits, CAD, total cholesterol levels, and metabolic syndrome (Kettunen et al., 2012; Zhou et al., 2013; van der Harst and Verweij, 2018).

As noted in Supplementary Table 5, several other variant-based associations were identified in the HNPT_EU that are not yet published as far as we are aware. For example, we observed a significant association between five *RASGRP3* variants and elevated triglycerides. The protein product, RAS Guanyl Releasing Protein 3, is a member of Ras guanyl-releasing family of proteins that are receptors for phorbol esters as well as diacylglycerol (DAG) (Stone, 2011). Importantly, the overproduction of DAG is associated with abnormal glucose metabolism (Hiramatsu et al., 2002; Das Evcimen and King, 2007), a published associated condition with elevated triglycerides (Parks, 2001). Additionally, the study identified nine variants in *DPP6* that were significantly associated with triglycerides. Previous studies suggest that *DPP6* binds specific voltage-gated potassium channels in neurons and plays a role in synaptic development and plasticity (Maussion et al., 2016; Lin et al., 2018). Interestingly, *DPP6* is predominantly expressed by pancreatic islets (Demine et al., 2020), suggesting a role in fatty acid metabolism. Indeed, previous studies have established a connection between *DPP6* and the glucose-insulin pathway, as reported by Imai et al. (2008). Two variants in *FOXO1* were also unpublished. Previous research demonstrates that *FOXO1*, a nuclear transcription factor, modulates the insulin response of apoC-III, a key enzyme influencing triglyceride metabolism (Altomonte et al., 2004).

### Gene-Based Association Analysis

Statistical power of classical rare single-variant GWAS is typically under-powered due to low minor allele frequencies and small sample sizes. Recent gene-based collapsing techniques such as SKAT, burden tests, and C-alpha address these issues by combining variants in a defined genetic region to increase statistical power. The SKAT method is ideal for our study as it (a) allows for combinations of common and rare variants via a weighting scheme (Lee et al., 2012); (b) it does not implicitly assume that all variants in one gene influence the trait in the same direction and same approximate magnitude as in the burden test; (c) it allows covariates to be included in the association model, unlike the C-alpha test (Wu et al., 2011). We observed variants within *APOC3* and within *GCKR* that are in proximity to each other (<10 kb) but have notably different effects on triglycerides in opposite directions. This is contrary to the main assumption behind a gene-based burden test (Wu et al., 2011).

We note here the utility of using gene-based association in tandem with single-variant association techniques. The advantage of this dual approach is especially pronounced in *APOC3*, found to have a strong gene-based association with triglyceride levels in the HNPT_EU, and one of the most-studied genes associated to triglyceride levels (Jørgensen et al., 2014; Hassan, 2014; TG and HDL Working Group of the Exome Sequencing Project et al., 2014; Kohan, 2015; Carey et al., 2016; Hu et al., 2016; Crawford et al., 2018; Reyes-Soffer et al., 2019). The gene collapse mechanism alone does not show that this gene-based association is driven by only two variants: rs138326449 and rs5128. The gene-based association (*p* = 2.65 × 10^–18^) is based on 18 variants. Without these two variants, the significance of the association is *p* = 0.02, rendering the gene-based association insignificant, genome-wide. Single-variant HNPT_EU analysis shows the strength of these associations (*p* = 5.35 × 10^–09^ and *p* = 2.08 × 10^–16^, respectively), as well as the notable difference in their effect magnitude and direction (β = −56.61, and β = 12.56, respectively). The variant rs5128 is a common variant that was previously associated with triglycerides in the UKB (Prins et al., 2017). Variant rs138326449 (R19X) is a well-studied rare loss-of-function variant with predicted ability to severely disrupt the function of Apolipoprotein C3, the protein product of *APOC3* (TG and HDL Working Group of the Exome Sequencing Project et al., 2014). Studies showed that this variant is associated with notably lower triglyceride levels; Jorgensen’s study reported a 39% decrease and a reduced risk of cardiovascular disease (Jørgensen et al., 2014; Carey et al., 2016). The HNPT_EU includes 42 heterozygotes (0.46%) whose mean triglyceride level is 51% lower than in those without the mutation (Supplementary Figure 2).

Another advantage of using both approaches is that SKAT identified five genes not observed in the single-variant association. One gene, *BAZ1B* was previously associated with triglycerides in several studies (Kathiresan et al., 2008; Johansen et al., 2010; Prins et al., 2017) and is hypothesized to be directly involved in lipid metabolism (Kong et al., 2015). Similarly, *PPM1G*, a gene whose protein product has been clinically verified to regulate the expression of *APOE* (Benson et al., 2018), demonstrates a strong association in the HNPT_EU cohort but does not contain significant single-variant associations. *APOE* is necessary in lipid metabolism and triglyceride-related responses to altered fat intake (Waterworth et al., 2010; Carvalho-Wells et al., 2012; Willer et al., 2013).

The gene-based association of *FNDC4* with triglycerides was not observed in the single-variant GWAS. *FNDC4*, which codes for Fibronectin Type III Domain-Containing Protein 4, functions as an anti-inflammatory factor on macrophages. (Bosma et al., 2016) and Lee et al. (2018) reported that *FNDC4* mitigates hyperlipidemia-induced insulin resistance through the suppression of inflammation and endoplasmic reticulum stress in adipocytes. PhenoScanner shows that this gene was previously associated with triglycerides based on the study by Willer et al. (2013), Staley et al. (2016), and Kamat et al. (2019) (Supplementary Table 6).

A gene-based association with *EIF2B4* was also undetected in the single-variant GWAS. This gene codes for the Eukaryotic Translation Initiation Factor 2B Subunit Delta, one of five subunits of the eIF2B complex. This complex is crucial for initiating the translation of mRNAs into peptides and therefore regulates the translation rate in the context of several different stress conditions (Rabouw et al., 2019; Marintchev and Ito, 2020). Variants in this gene have been associated with inappropriate insulin secretion from pancreatic β-cells (Bursle et al., 2019) and mutations in other subunits, which affect eIF2 signaling, have been associated with early onset diabetes (De Franco et al., 2020). Moreover, studies in animal models have connected eIF2 signaling to heart inflammation cardiac hypertrophy (Zhang et al., 2020). Willer et al. (2013) also found an association between this gene and triglycerides (Supplementary Table 6).

### Significant Phenotypic Associations With Triglyceride Levels and Pleiotropy

Elevated triglyceride levels were shown to be a significant predictor of expected phenotype groups (disorders of lipid metabolism, hyperlipidemia, and hypercholesterolemia, hypertension, ischemic heart disease, hypertensive heart disease) in the HNPT_EU, but also some less canonical clinical conditions, such as chronic liver and kidney diseases and several mood disorders. The odds ratios for these continuous-variable based associations are near one, indicating a small increase in absolute risk given a one-unit change in triglyceride levels; however, across the physical range of triglycerides, these differences are notable. The HNPT_EU odds ratio between triglycerides and chronic liver disease is approximately 0.3% (*p*-value < 4 × 10^–9^); although that implies only a 0.3% increase in the odds of developing chronic liver disease with a 1-mg/dl increase in triglyceride levels, it is notable that an individual with a triglyceride level of 150 mg/dl has a 10% increase in odds of developing chronic liver disease compared to a participant with the mean cohort level of 119 mg/dl, with respect to fixed covariates.

A number of established triglyceride-related variants listed in Supplementary Table 5 have been shown to exhibit pleiotropic effects in European cohorts with elevated C-reactive protein (Ridker et al., 2008; Dehghan et al., 2011; Ligthart et al., 2018), metabolic syndrome traits (Kraja et al., 2011), cholesterol levels (Hoffmann et al., 2018b), cardiovascular risk factors (Middelberg et al., 2011), coronary artery disease (Schunkert et al., 2011; Nelson et al., 2017), among others. These are quantitative trait studies, while the PheWAS is based on incidence of disease or condition as dictated by ICD codes. Our study does, however, identify pleiotropy in a number of variants. The variant rs77466627 is indicative of increased incidence of cardiac complications. Cerebral atherosclerosis also shows an increased incidence in the HNPT_EU in minor allele carriers of the variant rs137891079. A common variant, rs4938303, is found to be somewhat protective of GERD. It is of interest that many of the non-hyperglyceridemic associations have not yet been reported and may merit further examination in larger cohorts. As a majority of the variants tested in this study are indeed very rare, additional care must be carried out when examining associations. Although the Fisher test power calculation may add reassurance to the hypothesis test, all very rare variant hypotheses presented here should ultimately be carried out in larger cohorts.

### UK Biobank Validation

The UKB is a standard European cohort for common and rare variant analysis validation (Hoffmann et al., 2018a; Glentis et al., 2019; Cirulli et al., 2020; Siewert et al., 2020). As noted in the results, single-variant and gene-based associations identified in our study were validated to a great degree in the UKB: effect sizes and magnitudes, for the significant single-variants in both cohorts were, in most cases, very similar (Supplementary Table 7). The validation of the gene-based analysis was performed on an identical European cohort using the UKB exomes (Regier et al., 2018). Table 2 presents the gene-based validations. The two associations that could not be validated, *PPM1G* and *CSRNP2*, could be attributed to the differences in the cohort characteristics, or differences in the genotyping platforms: the UKB platform contains only exomes and exome adjacent variation (Van Hout et al., 2019), while the Helix Exome+ platform includes exomes and many other intronic and non-coding sites. In conclusion, the single-variant and gene-based GWAS results in the HNPT_EU were validated with strong significance in the UKB.

Using standard GWAS methods and gene-based association techniques with very rare, rare and common variants, coupled with a comprehensively EHR-cross-referenced cohort of notable size, our study validated a number of known gene-based and variant-based links with elevated triglycerides.

It also uncovered variants associated with elevated triglyceride levels that, to the best of our knowledge, are unpublished. Additionally, direct links between triglyceride levels and unexpected diseases in the HNPT_EU were exhibited. Pleiotropy of triglyceride-related variants revealed further associations yet unrecorded. We recognize that many variants reported here are very rare (and thus possibly unpublished) and will require future validation with larger cohorts. Although mostly academic in nature, the study, with its combination of approaches (single-variant GWAS, gene-based associations, phenotypic associations, and phenotype–genotype analyses), provides a powerful platform for the Healthy Nevada Project dataset. These are the first steps to explore a range of diseases and conditions, and to bridge bench and bedside with personalized translational medicine.

## Data Availability Statement

The data analyzed in this study is subject to the following licenses/restrictions: Genotype Data: These data are available to qualified researchers upon reasonable request and with permission of the Institute for Health Innovation and Helix. Researchers who would like to obtain the raw genotype data related to this study will be presented with a data user agreement which requires that no participants will be reidentified and no data will be shared between individuals or uploaded onto public domains. Due to the public nature of this article, and genetic privacy requirements, the Institute for Health Innovation and Helix require that the summary statistics of only 10,000 variants be made publicly available. This is the amount of data considered to be insufficient to enable a re-identification attack. The summary results of the most statistically significant 10,000 variants in this study are available here: https://www.dri.edu/renown-ihi/healthynvprojectgenetics/. Column definitions are provided in Supplementary Table 8. We attest that one author had full access to all the data in this study and takes responsibility for its integrity and the data analysis. The IHI encourages and collaborates with scientific researchers on an individual basis. Examples of restrictions that will be considered in requests to data access include but are not limited to: 1. Whether the request comes from an academic institution in good standing and will collaborate with our team to protect the privacy of the participants and the security of the data requested 2. Type and amount of data requested 3. Feasibility of the research suggested 4. Amount of resource allocation for the IHI and Renown Hospital required to support the collaboration Any correspondence and data availability requests should be addressed to JG at (Joe.Grzymski@dri.edu) or Craig Kugler (Craig.Kugler@dri.edu). EHR Data: EHR data for the Healthy Nevada Project cohort are subject to HIPAA and other privacy and compliance restrictions. The mean quality-controlled BMI and Triglyceride levels for each individual de-identified participant are available at https://www.dri.edu/renown-ihi/healthynvprojectgenetics/. Requests to access these datasets should be directed to JG, Joe.Grzymski@dri.edu; Craig Kugler, Craig.Kugler@dri.edu.

## Ethics Statement

The studies involving human participants were reviewed and approved by University of Nevada Institutional Review Board. The patients/participants provided their written informed consent to participate in this study.

## Author Contributions

RR and KS conceived the idea and experimental design of the study, performed data analysis and interpretation, and wrote the manuscript. JG provided funding and access to research components and principal investigator. VL provided scientific content to the manuscript. EC, NW, and JL provided access to crucial research components and revisions to the scientific content of manuscript. All authors contributed to the article and approved the submitted version.

## Conflict of Interest

JL, EC, and NW are employees of Helix Opco, LLC. The remaining authors declare that the research was conducted in the absence of any commercial or financial relationships that could be construed as a potential conflict of interest.

## Footnotes

1 ftp.ncbi.nlm.nih.gov/snp

2 https://www.ncbi.nlm.nih.gov/clinvar/

3 https://www.ebi.ac.uk/gwas/

4 https://cran.r-project.org/web/packages/SKAT/index.html

5 www.ukbiobank.ac.uk

## Abbreviations

HNPT_EU: Healthy Nevada Project triglyceride cohort
HER: electronic health record
CHD: coronary heart disease
LD: linkage disequilibrium
MAF: minor allele frequency
PCA: principal component analysis
VEP: Ensembl Variant Effect Predictor v.101
DM2: type II diabetes
PheWAS: phenome-wide association study
GWAS: genome-wide association study
CRP: C-reactive protein.
