# Supplementary material for: Genome-Wide Identification of Rare and Common Variants Driving Triglyceride Levels in a Nevada Population
Source: Front Genet. 2021 Mar 2;12:639418. doi: 10.3389/fgene.2021.639418 (PMC7982958; doi:10.3389/fgene.2021.639418)
Supplement: Supplementary file 1 [file Data_Sheet_1.PDF]

Supplementary Document 1: Computations showing that the power of the beta-coefficient in a linear regression is equivalent to the standardized beta-coefficient derived by standardizing the response variable into its z-score.

The mathematics showing that power of a standardized response variable and the resulting standardized effect size is equivalent to the power of a non-standardized response variable and its beta-coefficient.

The effect size of a single SNP against the dependent variable is measured here via the beta-coefficient β of the linear regression equation (ignoring all other covariates):

$y = \alpha + \beta x$, where x is the number of minor alleles that a person carries, and

$$\beta = \frac{n(\sum_{i=1}^{n} x_i y_i) - (\sum_{i=1}^{n} x_i)(\sum_{i=1}^{n} y_i)}{n(\sum_{i=1}^{n} x_i^2) - (\sum_{i=1}^{n} x_i)^2}$$

Substituting the standardized value $z_i = \frac{y_i - \bar{y}}{sd(y)}$ for each y_i (its z-score) yields the following:

$$\beta' = \frac{n\left[\sum_{i=1}^{n} x_i \left(\frac{y_i - \bar{y}}{sd(y)}\right)\right] - (\sum_{i=1}^{n} x_i)\left(\sum_{i=1}^{n} \frac{y_i - \bar{y}}{sd(y)}\right)}{n(\sum_{i=1}^{n} x_i^2) - (\sum_{i=1}^{n} x_i)^2}$$

$$= \frac{\frac{n}{sd(y)}(\sum_{i=1}^{n} x_i y_i - \sum_{i=1}^{n} x_i \bar{y}) - \frac{1}{sd(y)}(\sum_{i=1}^{n} x_i)(\sum_{i=1}^{n} y_i - \bar{y})}{n(\sum_{i=1}^{n} x_i^2) - (\sum_{i=1}^{n} x_i)^2}$$

$$= \frac{\frac{n}{sd(y)}(\sum_{i=1}^{n} x_i y_i - \sum_{i=1}^{n} x_i \bar{y}) - \frac{1}{sd(y)}(\sum_{i=1}^{n} x_i)(\sum_{i=1}^{n} y_i - n\bar{y})}{n(\sum_{i=1}^{n} x_i^2) - (\sum_{i=1}^{n} x_i)^2}$$

$$= \frac{\frac{n}{sd(y)}(\sum_{i=1}^{n} x_i y_i - \sum_{i=1}^{n} x_i \bar{y}) - \left(\frac{1}{sd(y)}[\sum_{i=1}^{n} x_i][\sum_{i=1}^{n} y_i] - (\sum_{i=1}^{n} x_i n\bar{y})\right)}{n(\sum_{i=1}^{n} x_i^2) - (\sum_{i=1}^{n} x_i)^2}$$

$$= \frac{\frac{n}{sd(y)}(\sum_{i=1}^{n} x_i y_i - \sum_{i=1}^{n} x_i \bar{y}) - \left(\frac{1}{sd(y)}[\sum_{i=1}^{n} x_i][\sum_{i=1}^{n} y_i] - n(\sum_{i=1}^{n} x_i \bar{y})\right)}{n(\sum_{i=1}^{n} x_i^2) - (\sum_{i=1}^{n} x_i)^2}$$

$$= \frac{\frac{n}{sd(y)}\sum_{i=1}^{n} x_i y_i - \frac{n}{sd(y)}\sum_{i=1}^{n} x_i \bar{y} - \frac{1}{sd(y)}\sum_{i=1}^{n} x_i \sum_{i=1}^{n} y_i + \frac{n}{sd(y)}\sum_{i=1}^{n} x_i \bar{y}}{n(\sum_{i=1}^{n} x_i^2) - (\sum_{i=1}^{n} x_i)^2}$$

$$= \frac{\frac{n}{sd(y)}\sum_{i=1}^{n} x_i y_i - \frac{1}{sd(y)}\sum_{i=1}^{n} x_i \sum_{i=1}^{n} y_i}{n(\sum_{i=1}^{n} x_i^2) - (\sum_{i=1}^{n} x_i)^2}$$

$$= \frac{\frac{1}{sd(y)}\left(n\sum_{i=1}^{n} x_i y_i - \sum_{i=1}^{n} x_i \sum_{i=1}^{n} y_i\right)}{n\left(\sum_{i=1}^{n} x_i^2\right) - \left(\sum_{i=1}^{n} x_i\right)^2}$$

$$= \frac{1}{sd(y)}\beta$$

Therefore, the regression $y = \alpha + \beta x$ can be rewritten as $z = \alpha + \frac{1}{sd(y)}\beta x$ where z denotes the standardized value of *y*, its z-score.

The power of the effect in the simple linear relationship $y = \alpha\ + \beta x$ is based on R^2, the coefficient of determination via Cohen's f^2 statistic:

$$f^2 = \frac{R^2}{1 - R^2},$$

with

$$R^2 = \frac{n\left(\sum_{i=1}^{n} x_i y_i\right) - \left(\sum_{i=1}^{n} x_i\right)\left(\sum_{i=1}^{n} y_i\right)}{\sqrt{\left[n\left(\sum_{i=1}^{n} x_i^2\right) - \left(\sum_{i=1}^{n} x_i\right)^2\right]\left[n\left(\sum_{i=1}^{n} y_i^2\right) - \left(\sum_{i=1}^{n} y_i\right)^2\right]}}.$$

Substituting the standardized value z for *y* yields:

$$R' = \frac{\frac{1}{sd(y)}\left(n\sum_{i=1}^{n} x_i y_i - \sum_{i=1}^{n} x_i \sum_{i=1}^{n} y_i\right)}{\sqrt{\left[n\left(\sum_{i=1}^{n} x_i^2\right) - \left(\sum_{i=1}^{n} x_i\right)^2\right]\left[n\left(\sum_{i=1}^{n} y_i^2\right) - \left(\sum_{i=1}^{n} y_i\right)^2\right]}},$$

using the above computations to simplify the numerator. Thus,

$$R' = \frac{\frac{1}{sd(y)}\left(n\sum_{i=1}^{n} x_i y_i - \sum_{i=1}^{n} x_i \sum_{i=1}^{n} y_i\right)}{\sqrt{\left[n\left(\sum_{i=1}^{n} x_i^2\right) - \left(\sum_{i=1}^{n} x_i\right)^2\right]\left[n\left(\sum_{i=1}^{n}\left(\frac{y_i - \bar{y}}{sd(y)}\right)_i^2\right) - \left(\sum_{i=1}^{n}\left(\frac{y_i - \bar{y}}{sd(y)}\right)\right)^2\right]}}$$

$$= \frac{\frac{1}{sd(y)}\left(n\sum_{i=1}^{n} x_i y_i - \sum_{i=1}^{n} x_i \sum_{i=1}^{n} y_i\right)}{\sqrt{\left[n\left(\sum_{i=1}^{n} x_i^2\right) - \left(\sum_{i=1}^{n} x_i\right)^2\right]\left[\frac{n}{sd(y)^2}\left(\sum_{i=1}^{n} y_i^2 - 2\bar{y}\sum_{i=1}^{n} y_i + n\bar{y}^2\right) - \left(\sum_{i=1}^{n} y_i - \frac{n\bar{y}}{sd(y)}\right)^2\right]}}$$

$$= \frac{\frac{1}{sd(y)}\left(n\sum_{i=1}^{n} x_i y_i - \sum_{i=1}^{n} x_i \sum_{i=1}^{n} y_i\right)}{\sqrt{\left[n\left(\sum_{i=1}^{n} x_i^2\right) - \left(\sum_{i=1}^{n} x_i\right)^2\right]\left[\frac{n}{sd(y)^2}\left(\sum_{i=1}^{n} y_i^2 - 2\bar{y}\sum_{i=1}^{n} y_i + n\bar{y}^2\right) - \frac{1}{sd(y)^2}\left(\sum_{i=1}^{n} y_i\right)^2 + 2n\bar{y}\sum_{i=1}^{n} y_i - n^2\bar{y}^2\right]}}$$

$$= \frac{\frac{1}{sd(y)}\left(n\sum_{i=1}^{n} x_i y_i - \sum_{i=1}^{n} x_i \sum_{i=1}^{n} y_i\right)}{\sqrt{\left[n\left(\sum_{i=1}^{n} x_i^2\right) - \left(\sum_{i=1}^{n} x_i\right)^2\right]\left[\frac{1}{sd(y)^2}\left(n\sum_{i=1}^{n} y_i^2 - 2n\bar{y}\sum_{i=1}^{n} y_i + n^2\bar{y}^2\right) - \frac{1}{sd(y)^2}\left(\sum_{i=1}^{n} y_i\right)^2 + 2n\bar{y}\sum_{i=1}^{n} y_i - n^2\bar{y}^2\right]}}$$

$$= \frac{\frac{1}{sd(y)}\left(n\sum_{i=1}^{n} x_i y_i - \sum_{i=1}^{n} x_i \sum_{i=1}^{n} y_i\right)}{\sqrt{\left[n\left(\sum_{i=1}^{n} x_i^2\right) - \left(\sum_{i=1}^{n} x_i\right)^2\right]\left[\frac{1}{sd(y)^2}\left(n\sum_{i=1}^{n} y_i^2 - 2n\bar{y}\sum_{i=1}^{n} y_i + n^2\bar{y}^2 - 1\left(\sum_{i=1}^{n} y_i\right)^2 + 2n\bar{y}\sum_{i=1}^{n} y_i - n^2\bar{y}^2\right)\right]}}$$

$$= \frac{\frac{1}{sd(y)}\left(n\sum_{i=1}^{n} x_i y_i - \sum_{i=1}^{n} x_i \sum_{i=1}^{n} y_i\right)}{\sqrt{\left[n\left(\sum_{i=1}^{n} x_i^2\right) - \left(\sum_{i=1}^{n} x_i\right)^2\right]\left[\frac{1}{sd(y)^2}\left(n\sum_{i=1}^{n} y_i^2 + n^2\bar{y}^2 - \left(\sum_{i=1}^{n} y_i\right)^2 - n^2\bar{y}^2\right)\right]}}$$

$$= \frac{\frac{1}{sd(y)}\left(n\sum_{i=1}^{n} x_i y_i - \sum_{i=1}^{n} x_i \sum_{i=1}^{n} y_i\right)}{\sqrt{\left[n\left(\sum_{i=1}^{n} x_i^2\right) - \left(\sum_{i=1}^{n} x_i\right)^2\right]\left[\frac{1}{sd(y)^2}\left(n\sum_{i=1}^{n} y_i^2 - \left(\sum_{i=1}^{n} y_i\right)^2\right)\right]}}$$

$$= \frac{\frac{1}{sd(y)}}{\frac{1}{sd(y)}} R^2$$

$$= R^2.$$

Thus, the coefficient of determination R^2 of the linear equation is unaltered upon the transformation.

And therefore the power computation for the standardized effect size $\beta' = \frac{\beta}{sd(y)}$ and the standardized variable $z = \frac{y-\bar{y}}{sd(y)}$ is equivalent to the one for the non-standardized effect size β and non-standardized variable y.

Supplemental Figures

Figure S1

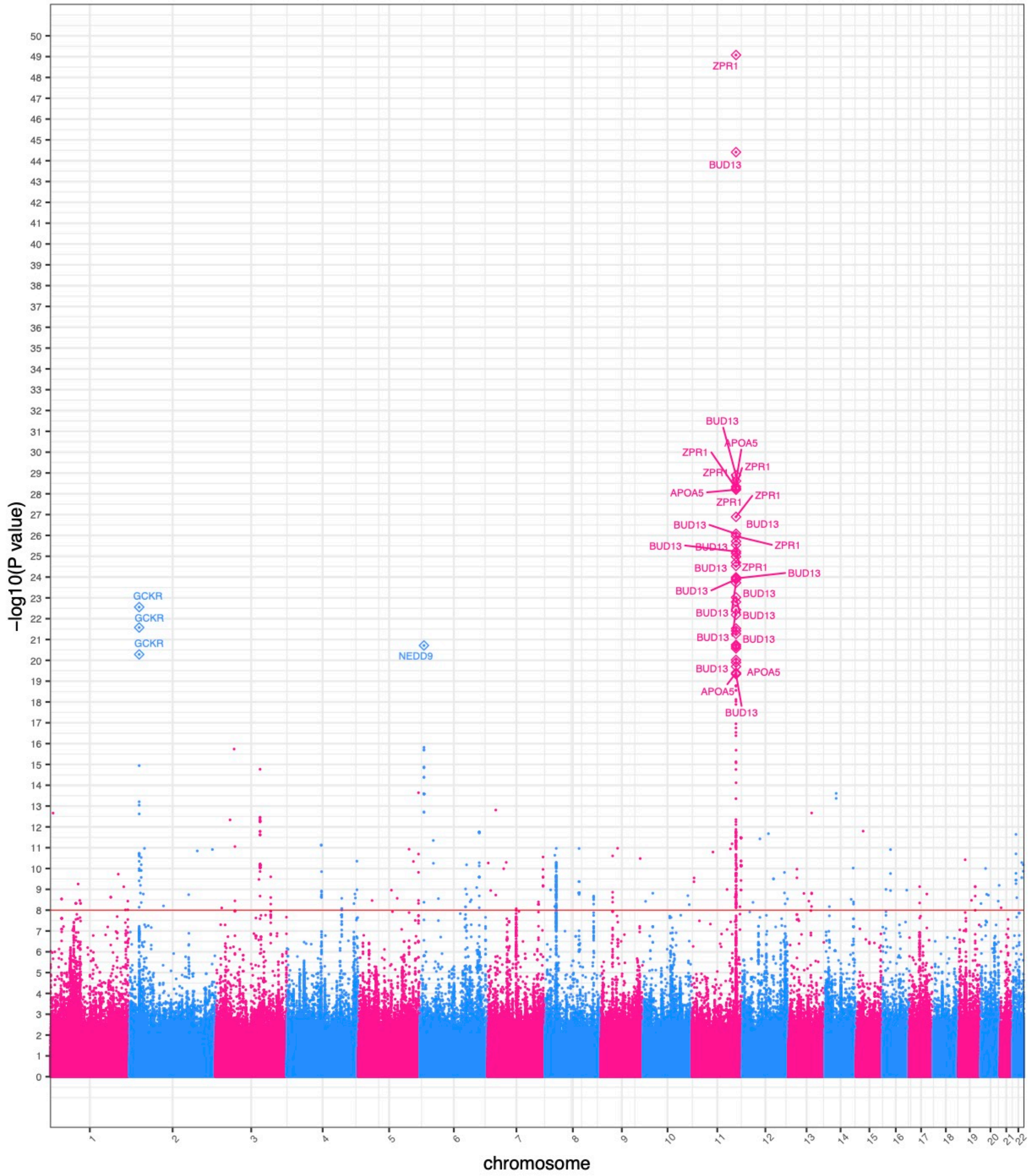

Each point in this figure represents a result of the single-variant genome-wide association study. The *x*-axis represents the genomic position of 5,712,318 variants. The *y*-axis represents -log$_{10}$-transformed raw *p*-values of each genotypic association. For ease of viewing, only variants within genes above the horizontal line $\alpha = 1x10^{-20}$, are annotated.

Figure S2

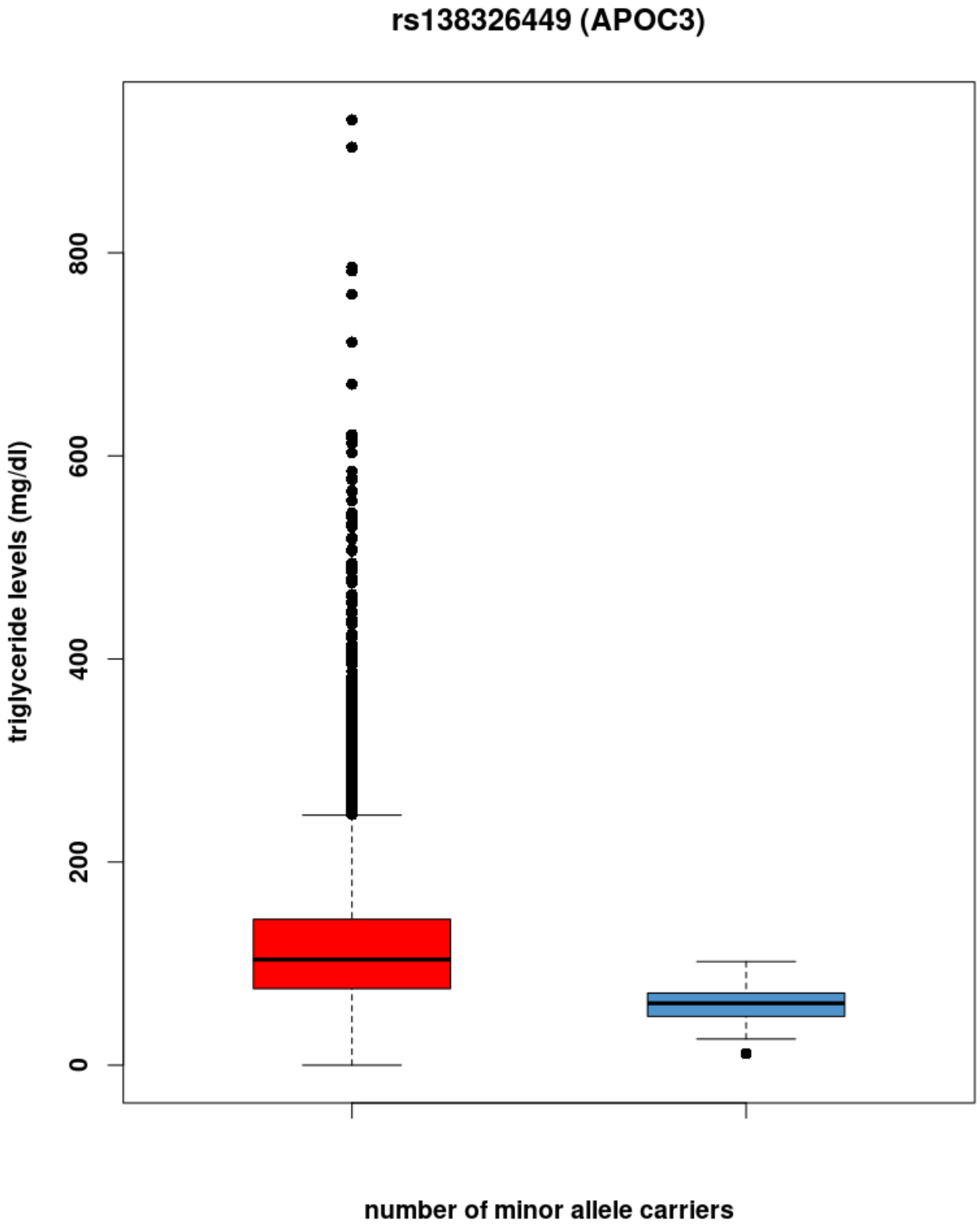

This figure shows differences in mean triglyceride levels (mg/dl) between sub-cohorts based on the number of copies of the minor allele of rs138326449 in *APOC3*. A simple non-parametric t-test between the triglyceride levels between the two sub-cohorts yields a raw *p*-value of p=3.7 x 10^{-15}. The mean ± standard deviation of the homozygotes in the reference allele is 119.3 ± 67.6 mg/dl; the mean ± standard deviation of the heterozygotes is 60.95 ± 19.15 mg/dl.

Supplemental Tables

Table S1. VEP annotation of statistically significant single variant associations

All single-variant associations were processed with the Ensembl Variant Effect Predictor v.101 algorithm to predict the functional consequences of each variant. For variants within genes, predicted functional consequences are listed for each transcript.

Table S2. Significant associations of phenotype groups with triglyceride levels

This table presents statistically significant associations of phenotype groups with triglyceride levels. Any association with *p*-value less than the adjusted significance level $\alpha = 3.85 \times 10^{-3}$ was deemed significant. The table includes the specific phenotype group description, the Beta-coefficient of the logistic regression (the effect that one mg/dl has on the incidence of the phenotype group), the odds ratio associated to one mg/dl change in triglyceride levels, the unadjusted *p*-value of the association, the number of cases, the number of controls upon application of exclusion criteria, and the broad phenotype group name.

Table S3: Analysis of 760,914 phenotype-genotype associations

This table shows results of 760,914 phenotype-genotype associations performed by a PheWAS to examine pleiotropy of 549 triglyceride-related variants of interest. Logistic models included the genotype, age, sex, BMI, DM2 diagnosis, antihyperlipidemic use, and the first four genotypic principal components. For each association in which the variant was a statistically significant predictor ($p < 5.0x10^{-5}$) of phenotype incidence, an additional Fisher exact test was performed to measure the direct association between variant and incidence of phenotype, without the covariates. The power of the Fisher allelic test, allelic odds ratio, and whether the association has been published are also included in the table.

Table S4. Demographics of the UKB Cohort

Table S4. Demographics of the UKB Cohort	
Cohort Size	35,321
Age (years)	67.91 ± 7.86
Male (%)	16,315 (46.19)
DM2	2,017 (5.71)
Antihyperlipidemic agents	6,406 (18.14)
Mean BMI Values	27.38 ± 4.75
Mean Triglyceride Values	1.71 ± 0.98 mmol/L
	(151.45 ± 86.80 mg/dL)

This table presents the demographics of the UKBB cohort used for replication. Summarized triglyceride units were converted by the standard conversion factor of 1mmol/L=88.57mg/dL, and simple properties of multiplication by constants in mean and standard deviation computations.

Table S5. Significant Single-variant HNP GWAS Associations

This table shows the results of the single-variant genome-wide association analysis. Variants are ordered with respect to chromosomal location. Genes and functional consequences were predicted by dbSNP 153 and PhenoScanner. Columns 3-5 denote whether the variant is published in PhenoScanner, ClinVar, or the GWAS catalog as associated with triglyceride levels. Beta indicates the effect size of the variant association, in mg/dl. P-values shown are unadjusted.

Table S6. Published Associations of Significant Gene Collapse Hits

All significant HNPT_EU gene collapse results were examined with PhenoScanner to identify previously published associations. Variants and genes are listed using rsID with GRCh38 coordinates. Studies presenting a published association are sorted by author and PubMed ID. Beta-coefficients, sample sizes, and *p*-values for each study are also listed.

Table S7. UKB Validation of HNP single-variant GWAS

This table presents independent validation of variants from chromosome 8 and 11 based on HNPT_EU GWAS. Significant variants from the HNPT_EU GWAS were compared to UKB imputed genotypes [Field ID 22801-22823]. Variants are ordered with respect to chromosomal location. Imputed variants from the UKB were lifted over from GRCh37 to GRCh38. UKB Beta-coefficients are shown as mmol/L as well as transformed to mg/dl. *P*-values shown are unadjusted.

Table S8. Column Identifiers for GWAS Results

Column name	Definition
CHR	Chromosome
SNP	Individual variant identifier
BP	Location of variant on relative chromosome in GRCh38
A1	Alternative Allele
TEST	Selected statistical test – ADD represents the additive effect
NMISS	Indicates the number of observations – non-missing genotypes
BETA	The effect size for this variant, defined per copy of the A1 allele
SE	The standard error of the effect size
LE	Lower end of the 95% confidence interval for the effect size
UE	Upper end of the 95% confidence interval for the effect size
STAT	The value of the test statistic
P	The p-value for the association test

Table describing the column headers for the results file of our genome-wide associations. This summary results file only lists the top 10,000 variants in order to prevent a re-identification attack. Data are found at https://www.dri.edu/renown-ihi/healthynvprojectgenetics/.
